# Surgical treatment of cerebellar pontine angle lipoma combined with trigeminal neuralgia: A case report

**DOI:** 10.1097/MD.0000000000041295

**Published:** 2025-01-17

**Authors:** Yu-Ting Yin, Chao Gui

**Affiliations:** a Department of Gastrointestinal Surgery, Hubei Cancer Hospital, Tongji Medical College, Huazhong University of Science and Technology, Wuhan, Hubei Province, China; b Department of Head and Neck Surgery, Hubei Cancer Hospital, Tongji Medical College, Huazhong University of Science and Technology, Wuhan, Hubei Province, China.

**Keywords:** case report, cerebellopontine angle lipoma, microvascular decompression, surgery, trigeminal neuralgia

## Abstract

**Rationale:**

Cerebellar pontine angle lipomas with trigeminal neuralgia are rare. The treatment choice is influenced by whether the pain is caused by the lipoma or the compression of blood vessels. Herein, we aimed to report a case of the disease and provide a reference for its treatment.

**Patient concerns:**

The patient was a 54-year-old female who presented with a 20-year history of left-sided facial pain. Her pain had gradually worsened over time and oral medications became progressively less effective.

**Diagnoses:**

Brain magnetic resonance imaging detected a left cerebellar horn lesion, which was deemed a lipoma. Three-dimensional time-of-flight magnetic resonance angiography revealed a superior cerebellar artery adjacent to the trigeminal nerve root. Trigeminal nerve roots may have been compressed by lipomas and blood vessels.

**Interventions:**

The patient underwent a microvascular decompression of the trigeminal nerve. Part of the lipoma was removed, and the trigeminal nerve was isolated from the blood vessels and tumor.

**Outcomes:**

Postoperative pathology confirmed a lipoma. Neuralgia was completely relieved postoperatively, and no new neurological disorder was detected during the 6-month follow-up.

**Lessons:**

Surgery is recommended for patients with cerebellar pontine angle lipomas combined with trigeminal neuralgia when conservative treatment fails. Detailed preoperative imaging is crucial to identify lipomas and trigeminal root compression by the responsible artery. Complete decompression of the trigeminal nerve root is necessary for complete pain relief.

## 1. Introduction

Intracranial lipomas are rare lesions accounting for 0.1% to 1.5% of all intracranial tumors and 0.14% of all cerebellopontine angle (CPA) tumors.^[1]^ Approximately 45% of intracranial lipomas occur in the interhemispheric fissure, and approximately 10% occur in the CPA.^[2]^ Although CPA lipomas may be incidentally detected because they can remain asymptomatic, they may become progressively symptomatic as they tend to wrap around the blood vessels and cranial nerves.^[3]^ Patients may experience hearing loss, vertigo, hemifacial spasms, facial sensory disorders, and trigeminal neuralgia (TN). Owing to surgical complications, conservative treatment is currently the mainstay of treatment, especially for slow-growing tumors and painless lesions, and frequent imaging is typically recommended to examine lesion growth. Carbamazepine and oxcarbazepine are first-line pharmacological treatments for TN. However, several patients experience side effects and those with persistent pain are unlikely to respond well to treatment.^[4]^ Surgical removal of CPA lipomas should be considered only in the presence of persistent or progressive symptoms or tumor growth.^[5]^

Herein, we report a case of CPA lipoma combined with TN in a patient who had been receiving TN treatment for 20 years. The patient experienced gradual medication failure and underwent surgical treatment involving resection of part of the lipoma tissue and microvascular decompression of the trigeminal nerve. After surgery, the patient experienced complete relief of TN, and no new neurological dysfunctions appeared.

## 2. Case report

A 54-year-old female presented with a 20-year history of maxillofacial pain in the left corner of the mouth, predominantly in V3 dermatomes. Initially, pain control was achieved using oral carbamazepine, oxcarbazepine, pregabalin, and painkillers. She had not undergone any cephalic head examinations until 4 years ago, when the pain had worsened, with brain magnetic resonance imaging (MRI) identifying lesions in the left CPA. To achieve pain control, the medication dosage was gradually increased. However, over the last 6 months, the patient’s condition progressively deteriorated, with frequent episodes of pain severely impairing her life. Physical examination revealed that she had percussion pain in the V3 dominant area of the left trigeminal nerve branch. The remaining neurological examinations revealed no abnormalities. The patient had no relevant medical history. She had no personal or family history of brain tumors or TN, and no history of other diseases. The results of routine complete blood count, kidney function, liver function, and coagulation function tests were normal, and no tumors were detected in other parts of the body.

**Figure 1.**
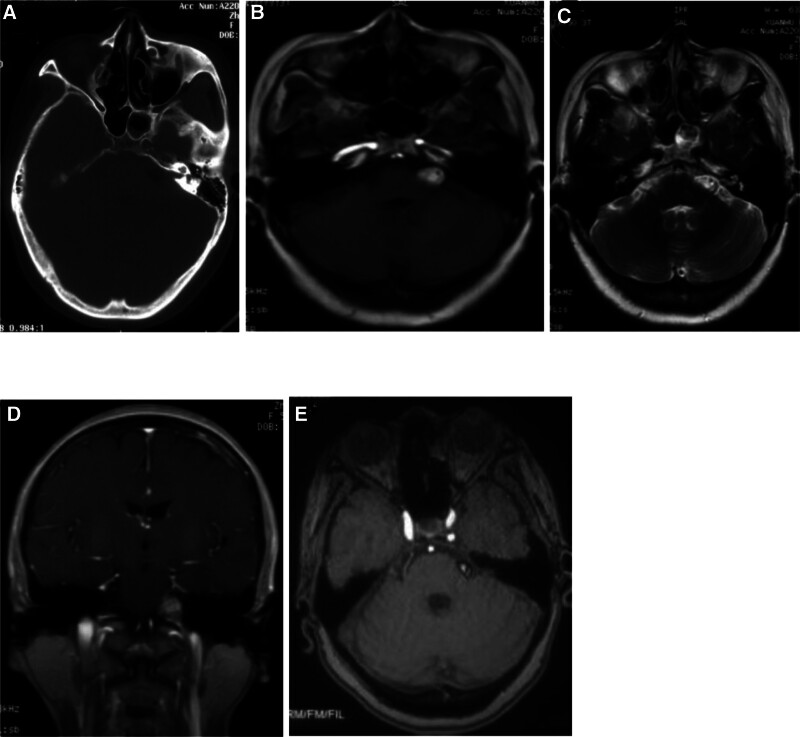
(A) Computed tomography scan of the head shows a hypodense lesion in the left cerebellopontine angle. (B) and (C) Magnetic resonance imaging showing a 1.3 × 0.9 cm lesion in the left cerebellopontine angle area. The mass shows high signal intensity on T1W1 and T2W1 images. (D) The lesion is not enhanced after gadolinium administration. (E) Three-dimensional time-of-flight magnetic resonance angiography showing that the left superior cerebellar artery is adjacent to the trigeminal nerve.

Computed tomography (CT) of the head revealed the presence of a hypodense lesion in the left CPA (Fig. 1A). Brain MRI revealed a 1.3 × 0.9 cm lesion in the left CPA region (Fig. 1B–D). The mass displayed a high signal intensity on T1W1 and T2W1 images, which was not enhanced after gadolinium administration. Diagnostic imaging suggested lipoma. MRI revealed that the lesion in the left CPA region was unaltered in terms of size over the last 4 years. Head 3-dimensional time-of-flight magnetic resonance angiography (3D-TOF-MRA) detected the superior cerebellar artery (SCA) adjacent to the trigeminal nerve root, and the left CPA tumor was closely related to the trigeminal and auditory nerves (Fig. 1E).

After careful consideration of the patient’s history, physical examination, laboratory tests, and imaging findings, we established a preliminary diagnosis of a left CPA lipoma combined with TN. The CPA lipoma and SCA compressed the trigeminal nerve.

As this was a persistent case and poor pain control was achieved with drug therapy, we decided to perform microvascular decompression of the TN via the posterior suboccipital sigmoid approach. The patient consented to undergo surgery.

## 3. Surgical intervention and outcomes

The specific surgical steps were as follows:

Step 1: After administering general anesthesia, the patient was placed in the prone position. A C-shaped incision was made behind the left ear, the scalp and occipital muscles were incised, the occipital bone was exposed, and a milling cutter was used to mill a bone window of approximately 3 × 3 cm in size; this bone window exposed the left transverse sinus and left sigmoid sinus.

Step 2: The dura mater was cut under the microscope, the dural flap was turned to the auricular side, and the arachnoid membrane was cut to release the cerebrospinal fluid from the greater occipital pool, followed by the collapse of the cerebellar tissue.

**Figure 2.**
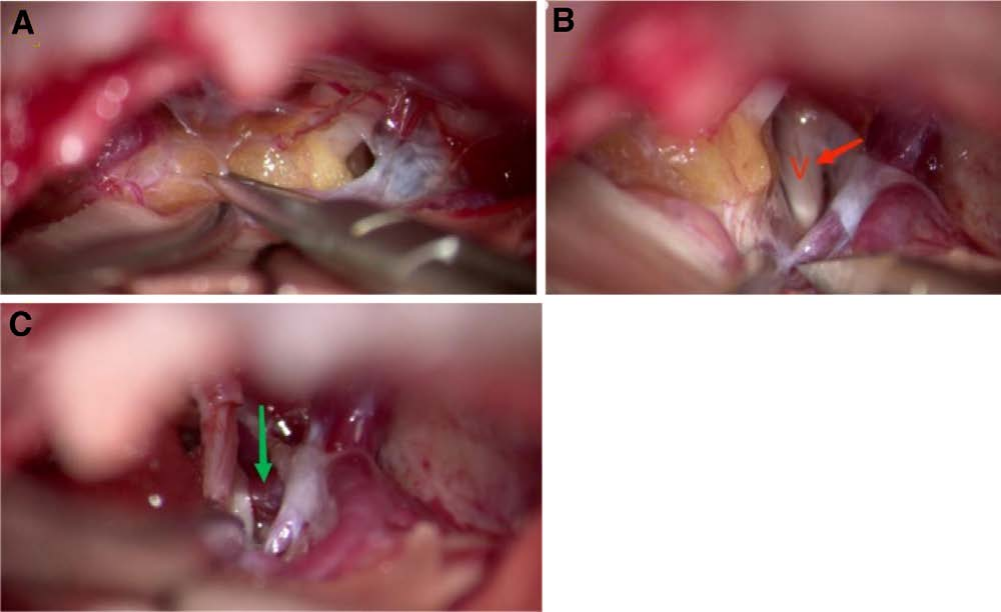
Position relationship of the cerebellopontine angle lipoma, trigeminal nerve, and superior cerebellar artery, as observed under the microscope. (A) Dissection of the arachnoid revealed lipomas enveloping the facial-auditory nerve complex. (B) The trigeminal nerve (red arrow) adjacent to the lipoma. (C) The superior cerebellar artery (green arrow) pressing upon the trigeminal root.

Step 3: Examination of the CPA revealed a mass of yellow adipose tissue encircling the facial-auditory complex (Fig. 2A), which was in close contact with the trigeminal nerve root (Fig. 2B).

Step 4: The arachnoid tissue surrounding the trigeminal nerve was clipped to reveal the cerebral pool segment of the trigeminal nerve from the root entry zone of the trigeminal nerve to Meckel’s cave. This revealed that the SCA was compressing the trigeminal nerve root from the medial measurements (Fig. 2C), whereas the CPA lipoma abutted the trigeminal nerve root on the lateral side.

**Figure 3.**
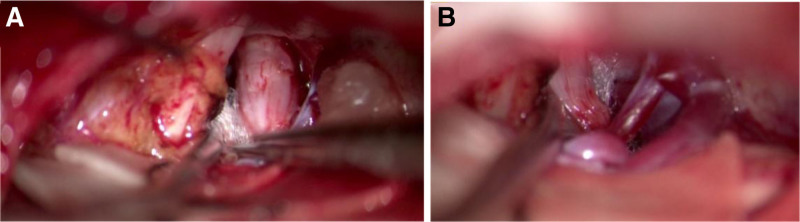
(A) After removing the lipoma tissue on the side adjacent to the trigeminal nerve, Teflon felt was placed between the lipoma and the trigeminal nerve. (B) Teflon felt separates the superior cerebellar artery from the trigeminal nerve.

Step 5: Part of the tumor tissue on the side close to the trigeminal nerve was excised to reduce the tumor size and relieve the compression of the trigeminal nerve root by the tumor. A Teflon felt was placed between the tumor and trigeminal nerve root (Fig. 3A) and between the trigeminal nerve root and SCA (Fig. 3B).

Step 6: The dura mater was tightly closed and the occipital musculature and skin were sutured.

Postoperatively, the patient’s facial pain was completely relieved, with no new neurological disorders. The pathological examination revealed small amounts of fat, fiber, and nerve fiber tissue, which confirmed the diagnosis. The patient was followed up for 6 months and experienced no further TN attacks.

## 4. Discussion

CPA lipomas are rare lesions representing 0.1% of all CPA tumors^[6]^; their combination with TN is even rarer. Intracranial lipomas are neither hamartomas nor true tumors but should be considered congenital malformations resulting from the abnormal persistence of the “meninx primitiva” during the development of the subarachnoid space pools and from the differentiation of lipomas.^[2]^ Most intracranial lipomas are asymptomatic and discovered incidentally. The signs and symptoms of CPA lipomas, including hearing loss (62%), dizziness (45%), TN, sensory impairment in the distribution of the fifth cranial nerve (14%), and facial dysfunction (9%), depend on the nerve structures of this region.^[7]^ Imaging techniques should differentiate it from vestibular schwannomas, meningiomas, arachnoid cysts, and epidermoids. CPA lipomas typically present as low-density masses on CT images. Lipomas are hyperintense on T1-weighted images, with a missing signal in the fat-suppression sequence, and not enhanced by gadolinium. The disappearance of the mass using fat-suppression techniques is specific to lipomas.^[1]^ With the widespread use of MRI, histopathological diagnosis is rarely required.^[8]^ Currently, the initial diagnosis is mainly based on MRI features. Our initial diagnosis of CPA lipoma was based on the CT and MRI images of the patient. The diagnosis was confirmed based on the appearance during surgery and final histopathological findings.

Classical TN (80–90%) is caused by the compression of the trigeminal nerve root by neighboring blood vessels.^[9]^ Tumors may be responsible for up to 5% of TN cases.^[10]^ Tumor compression of the trigeminal nerve leads to focal demyelination of the trigeminal root, triggering the same high-frequency discharges in the exposed axons as in vascular compression of the nerve.^[11]^ Typical TN usually presents with recurrent remitting pain accompanied by periods of complete pain relief, whereas atypical TN presents with persistent or sub-persistent pain.^[12]^ Secondary TN usually has no periods of inactivity. Detailed preoperative imaging is pivotal for identifying the underlying cause of TN. Fused 3D-TOF-MRA and 3-dimensional constructive interference in steady-state images are reliable, noninvasive tools for evaluating diseased vessels and disease extent in patients with neurovascular compression.^[13]^ This patient underwent a comprehensive 3D-TOF-MRA examination, revealing that the SCA and CPA lipomas were adjacent to the trigeminal nerve root. Intraoperative treatment of blood vessels and tumors is required for complete relief of TN.

Lipomas grow slowly. For instance, Totten et al^[14]^ did not observe persistent lipoma growth in 17 patients over a mean follow-up period of 47 months. However, lipomas typically entangle cranial nerves and are highly vascularized; hence, complete surgical removal can be challenging, and even patients who have undergone incomplete excision or simple biopsy may experience serious sequelae postsurgery.^[15]^ Considering its slow or lack of growth, conservative treatment is the first option. Carbamazepine and oxcarbazepine, both anticonvulsant agents, are considered the treatment of choice for controlling paroxysmal pain in patients with TN, regardless of the etiology. These drugs elicit effective pain relief in almost 90% of patients.^[16]^ However, clinical improvements are frequently offset by side effects such as dizziness, diplopia, ataxia, and elevated transaminase levels, 1 or more of which may result in 23% of patients discontinuing treatment.^[17]^ In this case, our patient had TN for 20 years, which was initially manageable with medication. However, even with the gradual increase in medication dosage, the pain became progressively difficult to control.

Although surgery is the most invasive technique for treating TN, it offers the lowest pain recurrence rate and highest patient satisfaction.^[18]^ One challenge encountered during this study was the intraoperative removal of the lipoma, which wrapped around the facial auditory nerve and contained abundant tiny blood vessels, making intraoperative hemostasis difficult and risking facial auditory nerve injury. Surgical removal of CPA lipomas may lead to serious consequences, as the tumor tends to attach to nerves, vascular structures, and surrounding tissues.^[19]^ Complete removal of lipomas can be complicated, and even patients who undergo incomplete resection or simple biopsy may experience severe postoperative neurological deficits. Dudulwar et al^[20]^ reported that non-radical resection can result in hearing loss and grade 4 facial palsy.

In the present case, after establishing that the lesion was a lipoma and that the SCA was adjacent to the trigeminal nerve, surgery was performed to address the patient’s progressively deteriorating symptoms.

Intraoperatively, it was confirmed that the lipoma and SCA compressed the trigeminal nerve root. Lipomas located lateral to the trigeminal nerve root restrict the movement of the nerve root. Although the lipoma encapsulated the facial-auditory nerve complex, no associated symptoms were observed. Instead of complete tumor resection, partial resection was performed to reduce the tumor size. Teflon felt was placed on both sides of the trigeminal nerve root to relieve the compression induced by the tumor and blood vessels. The thickened arachnoid membrane enveloping the trigeminal nerve was cut to loosen the pull on the trigeminal nerve, and decompression of the entire trigeminal nerve in the cerebral pools was performed, which considerably increased the activity of the trigeminal nerve. The TN was completely relieved postoperatively, and other neurological examinations were unaltered. The patient recovered well after the surgery and did not require oral medication.

Thus, when conservative treatment for a cerebellar pontine angle lipoma combined with TN is ineffective, trigeminal nerve root surgery is recommended to decompress the trigeminal nerve root. Protecting neurological function is important when resecting a lipoma, and as there are often nerves and blood vessels penetrating the lipoma, total excision of the lipoma should be avoided to prevent new neurological dysfunctions.

This study has some limitations. Firstly, this single case cannot be considered generalizable. Secondly, although the 6-month follow-up showed no signs of symptom recurrence, the long-term effects of this treatment still need further investigation.

## 5. Conclusion

The coexistence of CPA lipomas and TN is a rare phenomenon, and nonsurgical conservative treatment is preferred. However, surgery should be considered in cases of progressively worsening pain and poor management with conservative treatment. Detailed preoperative MRI and 3D-TOF-MRA are crucial for identifying the primary cause of TN. The goal of surgery is not to completely remove the lipoma but completely relieve trigeminal nerve compression. In the current case, compression of the SCA was identified as the underlying cause of TN, in addition to the CPA lipoma tumor, and surgical release of compression resolved the patient’s facial pain.

## Acknowledgments

We express our gratitude to all those who participated in this study.

## Author contributions

**Conceptualization:** Yu-Ting Yin, Chao Gui.

**Data curation:** Yu-Ting Yin.

**Formal analysis:** Chao Gui.

**Investigation:** Yu-Ting Yin.

**Methodology:** Chao Gui.

**Supervision:** Chao Gui.

**Validation:** Chao Gui.

**Writing – original draft:** Yu-Ting Yin, Chao Gui.

**Writing – review & editing:** Yu-Ting Yin, Chao Gui.
